# Establishment and Prospective Validation of a Classification System for Surface Microvessels in Peritoneal Lesions Under Magnifying Endoscopy

**DOI:** 10.3390/diagnostics16182984

**Published:** 2026-09-15

**Authors:** Xianwen Guo, Liqi Shen, Ronge Lei, Pengyu Huang, Jiao Li, Zhen Ding, Rong Lin

**Affiliations:** 1 Endoscopy Center, The First Affiliated Hospital, Sun Yat-sen University, No. 58 Zhongshan Er Road, Guangzhou 510080, China; guoxw26@mail.sysu.edu.cn; 2 Department of Gastroenterology, Union Hospital, Tongji Medical College, Huazhong University of Science and Technology, 1277 Jiefang Avenue, Wuhan 430022, China; 3 Department of Gastroenterology, The People’s Hospital of Guangxi Zhuang Autonomous Region, Nanning 530021, China; 19167510890@163.com (L.S.); guoxiang@163.com (P.H.); 19317199936@163.com (J.L.); 4 Department of Gastroenterology, First Affiliated Hospital of Guangxi Medical University, Nanning 530021, China; 17377527099@163.com

**Keywords:** magnifying endoscopy, peritoneal, precision, diagnosis, natural orifice transluminal endoscopic surgery

## Abstract

**Background:** Peritoneal nodules require abdominal exploration and biopsy. However, blind biopsies may yield false-negative results. We developed a magnifying endoscopy-based microvascular classification for peritoneal lesions, prospectively validated its pathological predictive accuracy, and explored a precision-targeted biopsy strategy. **Methods:** This two-phase study was conducted at three hospitals from June 2016 to May 2022 (retrospective classification development) and four hospitals from June 2022 to February 2023 (prospective validation). Two experienced endoscopists first extracted features independently under blinded conditions and then constructed the microvascular classification (Types 1–3D) through an unblinded iterative process. Retrospective patients undergoing white-light endoscopic exploration served as non-randomized controls. Diagnostic accuracy, biopsy number, and histological diagnostic yield were compared. **Results:** This retrospective cohort included 56 patients with 77 peritoneal lesions. Of the 29 prospectively enrolled patients, 25 patients with 76 peritoneal lesions were included in the final validation. Three out-of-scope cases were reported separately. At the patient level, magnifying endoscopy showed significantly higher accuracy in predicting lesion pathology than white-light endoscopy (83.93% vs. 46.00%, *p* < 0.001), with fewer biopsies (*p* = 0.002) and a higher histological diagnostic rate (*p* < 0.001). Prospectively, the classification accuracy was 96.05% for tuberculosis (Type 2C), 96.05% for tumor-like lesions (Type 3A), 96.05% for mesothelioma (Type 3B), 97.37% for Müllerian epithelial tumors (Type 3C), and 90.79% for metastatic carcinoma (Type 3D). **Conclusions:** The microvascular classification showed promising preliminary accuracy for predicting peritoneal lesion pathology and may support precision-targeted biopsy. External validation and formal reproducibility assessment are required before clinical implementation.

## 1. Introduction

Peritoneal nodules are a common clinical finding, with lesions typically identified on CT, MRI, and other imaging examinations. However, these imaging methods cannot obtain tissue samples for pathological diagnosis [1]. Diagnosis primarily relies on abdominal exploration and biopsy to obtain tissue samples for pathological evaluation. During surgery, the presence of diffuse peritoneal lesions of varying sizes necessitates blind biopsy based on the operator’s experience, which can lead to false-negative pathological results and subsequent misdiagnosis and mistreatment [2,3]. A study showed that the sensitivity, specificity, and accuracy of laparoscopic biopsy for peritoneal lesions were 47.8%, 85.7%, and 62.2%, respectively [2]. Therefore, new methods and criteria are needed to enhance biopsy accuracy and the positive rate of pathology.

Novel magnifying endoscopy imaging techniques utilize blue and green light. Due to the high absorption of these wavelengths by hemoglobin, the shape of blood vessels can be clearly displayed [4,5,6]. Under narrow blue- and green-light illumination, this technique magnifies images by more than 100 times, allowing detailed observation of superficial microvasculature and microstructure. Recent reports highlight the effectiveness of novel magnifying endoscopy in identifying and assessing gastrointestinal tumors [7,8,9,10,11]. Although the peritoneal surface and digestive tract mucosa differ in histological origin, their surface microvascular optical imaging characteristics are similar under pneumoperitoneum; thus, magnifying endoscopy is theoretically applicable to peritoneal lesion assessment. However, the application of magnifying endoscopy to peritoneal lesions and the diagnostic value of its microvascular features have not been established. This study aimed to: (1) establish a microvascular classification standard for peritoneal lesions based on magnifying endoscopy; (2) prospectively validate the accuracy of this standard in predicting lesion nature; and (3) explore precise biopsy strategies based on this standard, with the goal of improving the accuracy and histological diagnosis rate of peritoneal lesion biopsy.

## 2. Methods

### 2.1. Study Design

**Figure 1:**
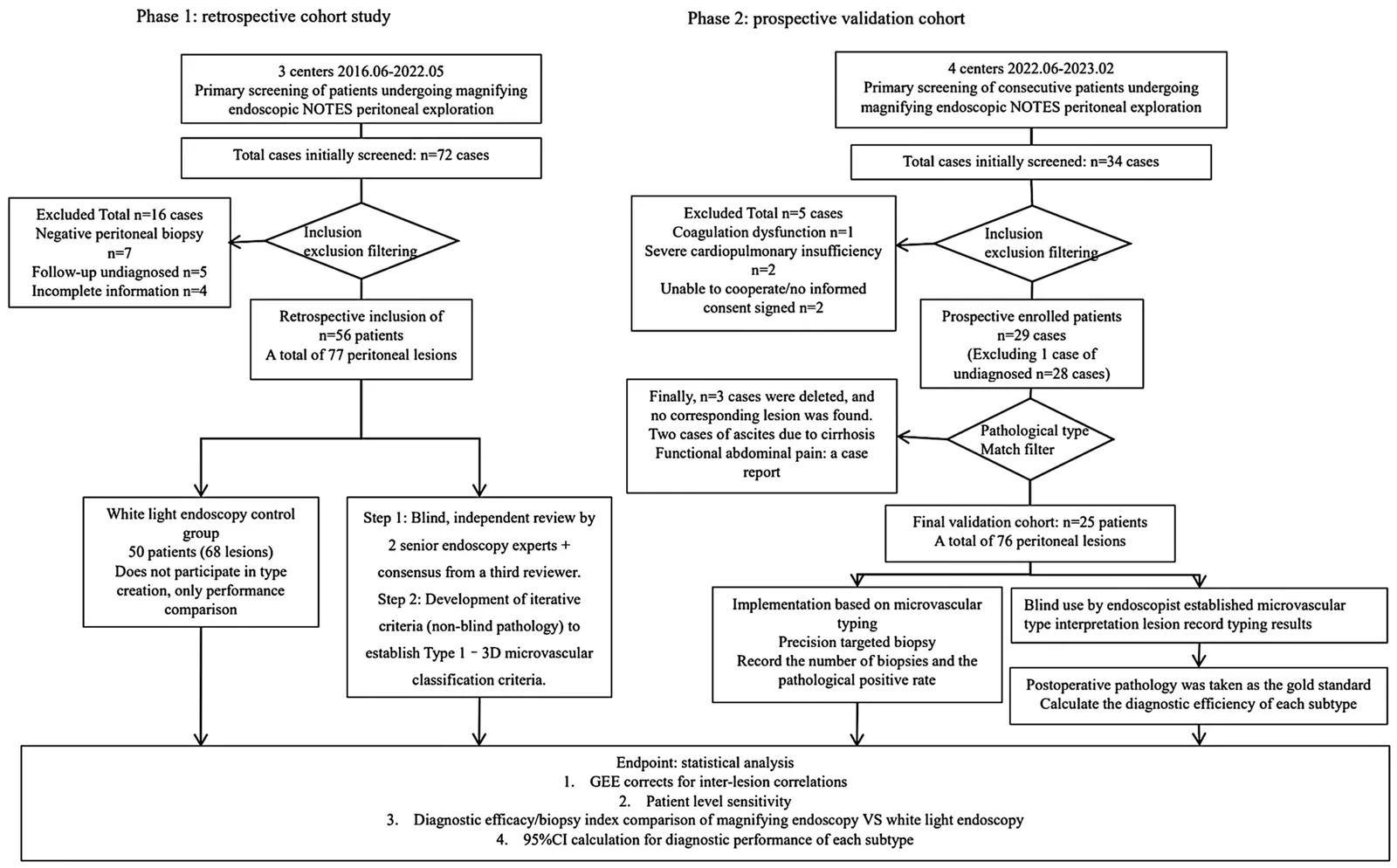
Study flow diagram.

This study was conducted in two phases. The first phase was a retrospective study (June 2016 to May 2022) that collected clinical data and images from patients who underwent magnifying endoscopic NOTES peritoneal exploration at People’s Hospital of Guangxi Province, People’s Hospital of Pingxiang, and Wuhan Union Hospital, for the development of the microvascular classification standard. The second phase was a prospective diagnostic accuracy study (June 2022 to February 2023) that enrolled patients undergoing the same examination at People’s Hospital of Guangxi Province, Wuhan Union Hospital, People’s Hospital of Pingxiang, and Hechi People’s Hospital of Guangxi Province, to prospectively validate the classification standard (Figure 1).

### 2.2. Patients

For the retrospective cohort, inclusion criteria were: (1) patients who underwent magnifying endoscopic NOTES peritoneal exploration and biopsy; (2) patients with biopsy-confirmed diagnoses; (3) an initial diagnosis consistent with the diagnosis during follow-up; and (4) complete magnifying endoscopy images. Exclusion criteria were: (1) patients with negative results on all peritoneal biopsies; (2) patients not diagnosed by the end of follow-up; and (3) incomplete clinical information.

For the prospective cohort, inclusion criteria were: (1) patients scheduled for NOTES peritoneal exploration and biopsy due to peritoneal lesions; (2) age 18–80 years; and (3) provision of written informed consent. Exclusion criteria were: (1) coagulation disorders; (2) severe cardiopulmonary insufficiency; (3) pregnancy; and (4) inability to cooperate with the examination or provide informed consent.

This study was approved by the Ethics Committee of Tongji Medical College, Huazhong University of Science and Technology (Approval No. [2020] S322, Approval date: 25 November 2020), and all patients provided written informed consent.

### 2.3. Procedure for Magnifying Endoscopic Peritoneal Exploration and Biopsy

After general anesthesia, the trans-umbilical or trans-gastric approach was selected according to the lesion location to establish pneumoperitoneum. The sterilized magnifying endoscope was inserted into the abdominal cavity through the puncture channel. The entire abdominal cavity was first examined in white-light mode to observe the peritoneal condition and identify suspicious lesions. The lesions were centered in the endoscopic field, and long-, medium-, and close-range white-light images were captured sequentially. The endoscope was then switched to blue laser imaging (BLI) or narrow-band imaging (NBI) mode, gradually approaching the lesions to capture low-, medium-, and high-magnification images. Observation was performed first at the edge of the lesion, followed by the center. Precise biopsies of suspicious lesions were conducted under real-time endoscopic monitoring.

### 2.4. Biopsy Procedure

In BLI or NBI combined with low- and medium-magnification modes, a target region was selected. Biopsy forceps were extended, and the endoscopic mode was kept unchanged. Biopsy was performed under real-time monitoring. Only one sample was obtained from lesions with a diameter <1 cm, and 2–3 samples were obtained from lesions with a diameter >1 cm. All biopsy samples of each lesion were placed into the same bottle, and the location was marked. The next lesion was observed and accurately biopsied using the same method. The final pathological diagnosis was confirmed by at least two independent senior pathologists.

### 2.5. Establishment of the Microvascular Classification Standard

#### 2.5.1. Independent Feature Extraction (Blinded Phase)

Prior to classification standard development, two endoscopists with more than 10 years of experience in magnifying endoscopy (Xianwen Guo, Rong Lin) independently evaluated all magnifying endoscopic images in the retrospective cohort. The two endoscopists case-by-case recorded the morphological features, regularity, vessel diameter, branching, density, distribution, arrangement, and brown spots of the surface microvessels of each lesion. Both evaluators remained blinded to the final pathological diagnosis during this phase.

#### 2.5.2. Consensus Feature Description (Still Blinded)

For cases with inconsistent independent interpretations, a third endoscopist (Zhen Ding) was invited to participate in the discussion to reach consensus, forming preliminary feature descriptions. This phase remained blinded to pathological diagnosis.

#### 2.5.3. Standard Construction (Unblinded Iterative Phase)

After feature extraction and consensus description were completed, the final pathological diagnoses were uniformly revealed. An iterative method was used to compare the extracted microvascular features with pathological results: first, microvascular patterns repeatedly associated with specific pathological types were identified; preliminary types were defined; then, through repeated revision and internal validation, a stable classification standard was formed. Ultimately, the surface microvessels of peritoneal lesions were classified into Type 1, Type 2 (2A, 2B, 2C), and Type 3 (3A, 3B, 3C, 3D).

#### 2.5.4. Note on Development-Phase Performance Estimation

Notably, the above iterative construction process used pathological results from the derivation cohort to optimize the typing standard. Therefore, the “diagnostic accuracy” reported in the retrospective derivation cohort is essentially an internal consistency validation during the classification development phase, rather than an independent diagnostic accuracy estimate. The true independent diagnostic performance assessment of this study comes solely from the prospective validation cohort in phase 2.

### 2.6. White-Light Endoscopy Control

In the retrospective cohort, 50 patients who underwent white-light endoscopy (non-magnifying) peritoneal exploration served as the control group. Their lesion nature prediction, number of biopsies, and pathological positive rate were compared with those of 56 patients who underwent magnifying endoscopy during the same period. It should be emphasized that this control was not generated through randomization or paired matching but was based on concurrent clinical pathway selection at the same hospitals (physicians selected the endoscopic mode according to equipment availability and clinical judgment). Baseline characteristics (age, sex, BMI, lesions per patient) and pathological type distributions of the two groups are shown in Supplementary Tables S1 and S2.

### 2.7. Proposed Strategy for Guiding the Accurate Biopsy Based on the Microvascular Classification Criteria

Based on the microvascular classification standard, corresponding precise biopsy strategies were formulated: (1) Type 1, 2A, and 2B represent normal peritoneum, acute peritonitis, and chronic peritonitis, respectively; current evidence is insufficient to support an absolute clinical decision of “no biopsy required”; biopsy necessity should be carefully evaluated in strictly selected cases until external validation and inter-observer reproducibility have been confirmed; (2) for Type 2C (tuberculosis), biopsies should be performed in both the central avascular region and the marginal vascular region; (3) for Type 3A (tumor-like lesions), biopsy should be performed in the central region; (4) for Type 3B (mesothelial tumors) and Type 3C (Müllerian epithelial tumors), biopsies should be performed in areas with dense microvessels; (5) for Type 3D (metastatic carcinoma), multiple deep biopsies (≥3 times) should be performed in areas with irregular microvessels.

### 2.8. Statistical Analysis

All statistical analyses were performed using SPSS software (version 22.0). Normally distributed continuous data are expressed as mean ± standard deviation (SD) and compared using Student’s *t*-test, analysis of variance, or Mann–Whitney U test. Categorical variables were compared using chi-square analysis or Fisher’s exact test, as appropriate. Differences in *p* < 0.05 were considered statistically significant.

This study involved dual analytical units at both the patient level and the lesion level. For lesion-level comparisons (such as the accuracy of predicting lesion nature under the two endoscopic modes), since the same patient might carry multiple lesions of the same pathological type, the data exhibited within-group correlation. Therefore, generalized estimating equations (GEE) were used for correction, with patient ID as the cluster variable, specifying an exchangeable correlation structure to correct for within-group correlation of multiple lesions in the same patient. For patient-level sensitivity analysis, only 17 patients who simultaneously completed paired examinations with both magnifying endoscopy and white-light endoscopy during the same period were included. Data were aggregated according to the strict criterion that “if any lesion in a patient was predicted incorrectly, the patient was counted as incorrectly predicted.” Because this was a paired design, McNemar’s test was used to compare patient-level accuracy between the two modes.

Diagnostic performance indicators (sensitivity, specificity, positive predictive value [PPV], negative predictive value [NPV], and accuracy) and their 95% confidence intervals (95% CI) were calculated using the Clopper–Pearson exact method. Particularly, these confidence intervals were calculated at the lesion level, assuming each lesion was an independent observation; because the same patient might carry multiple lesions, the data exhibited within-group correlation. Therefore, these intervals should be interpreted as descriptive references rather than strict statistical inference boundaries. Comparisons of diagnostic accuracy between the two modes were performed using the chi-square test for unpaired patient-level and lesion-level data, McNemar’s test for the paired patient-level sensitivity analysis, and GEE for the clustered lesion-level analysis. *p* < 0.05 was considered statistically significant.

## 3. Results

### 3.1. Patient Characteristics

The retrospective cohort included 56 patients with 77 peritoneal lesions, including nine lesions of chronic peritonitis, 20 of tuberculosis, 12 of tumor-like lesions, 7 of Müllerian epithelial tumors, 7 of mesothelial tumors, and 22 of metastatic carcinoma. The prospective validation cohort consecutively enrolled 29 patients. One patient was excluded due to lack of pathological confirmation. Of the 28 patients who completed the examination and were included in the study, 25 patients with 76 peritoneal lesions were included in the final analysis. The final analysis included 19 tuberculosis lesions, 13 tumor-like lesions, 12 mesothelial tumors, 6 Müllerian epithelial tumors, and 26 metastatic carcinomas. Another three patients (2 with cirrhotic ascites and one with functional abdominal pain) were not included in the final analysis because their pathological results did not fall within the lesion types covered by this microvascular classification; their clinical information is reported separately in the Discussion.

#### Baseline Comparability of the Two Retrospective Groups

There were no statistically significant differences between the two groups in age, sex, BMI, lesions per patient, or overall pathological type distribution (all *p* > 0.05) (Supplementary Tables S1 and S2). However, it should be noted that this control was not generated through randomization or paired matching but was based on concurrent clinical pathway selection at the same hospitals (physicians selected the endoscopic mode according to equipment availability and clinical judgment); therefore, potential selection bias cannot be excluded. The average number of lesions per patient was comparable between the two groups (1.4 ± 0.8 vs. 1.4 ± 0.7; *p* = 0.89) (Supplementary Tables S1 and S2). The underlying raw means were 1.38 and 1.36, respectively; the difference in total lesion count (77 vs. 68) was primarily attributable to the different sample sizes (56 vs. 50 patients) rather than a systematic difference in lesion burden per patient.

### 3.2. Characteristics of Different Peritoneal Lesions Under the Magnifying Endoscopy

**Figure 2:**
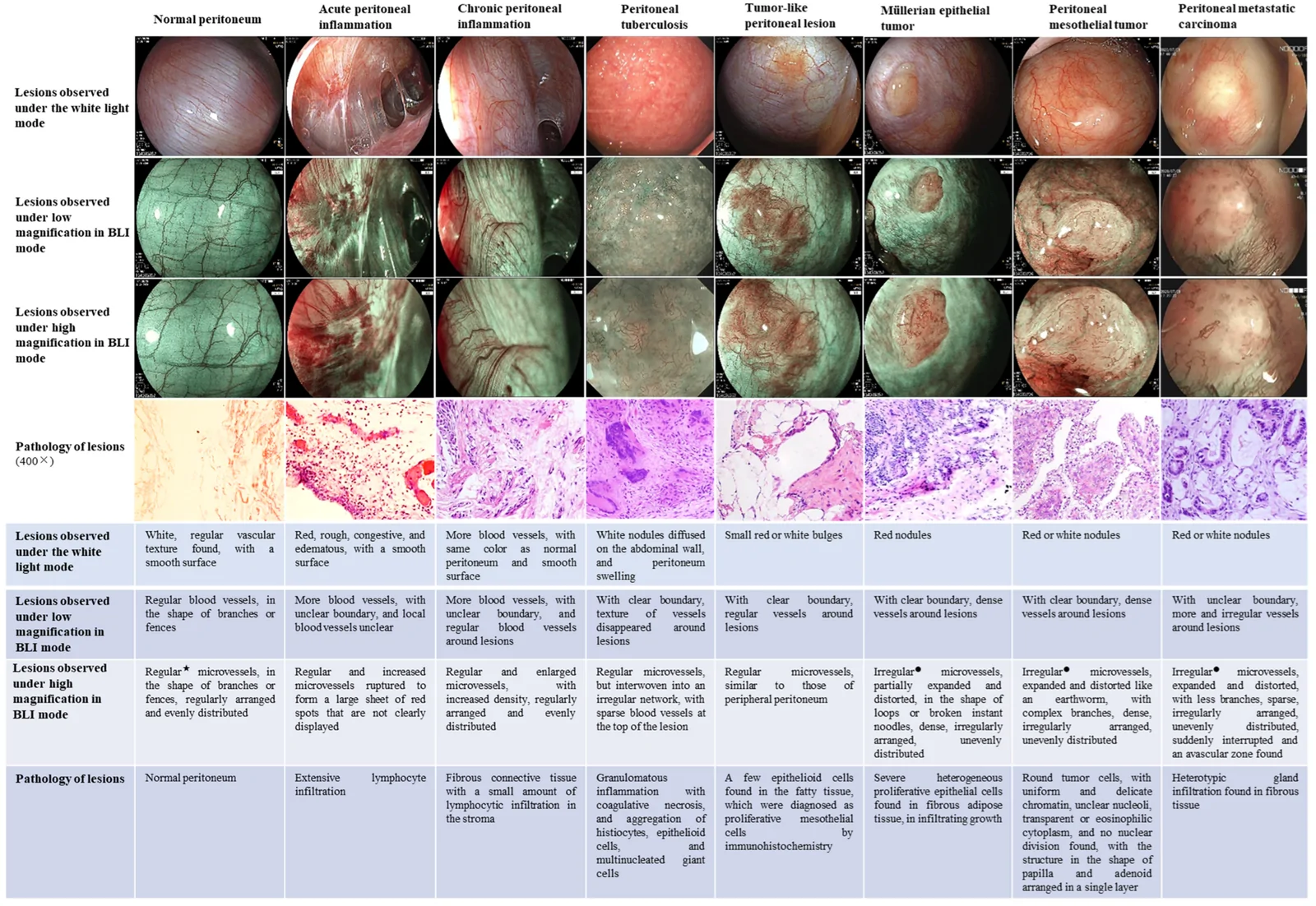
Characteristics of different peritoneal lesions under magnifying endoscopy. ^★^ Regular: Microvessels have a similar shape with uniform diameter; ^●^ Irregular: Microvessels have various shapes with diameter inconsistency, abnormal expansion (>1.5× normal).

Under magnifying endoscopy, significant differences were observed among different lesions in terms of shape (Paris classification [12]), color, boundary, diameter, surface smoothness, peripheral vascular features, and surface microvascular characteristics (*p* < 0.05). The microvessels of chronic inflammatory lesions and tuberculous lesions were mostly regular in morphology, with uniform diameter and orderly arrangement, whereas the microvessels of Müllerian epithelial tumors, mesothelial tumors, and metastatic carcinoma were irregular, with inconsistent diameter, abnormal expansion (>1.5 times normal diameter), complex branching, abnormally increased density, irregular arrangement, and uneven distribution (*p* < 0.05). Furthermore, significant differences were noted among all lesion categories in terms of surface microvascular visibility, regularity, abnormal expansion, abnormal distortion, shape consistency, diameter consistency, branching complexity, density, sparseness, arrangement, distribution, choke vessels, and brown spots (*p* < 0.05) (Table 1 and Figure 2).

### 3.3. Comparison of the Effectiveness of Common White-Light Endoscopy and Magnifying Endoscopy in Diagnosing the Nature of Peritoneal Lesions

This comparison involved two analytical units. At the patient level (unit of analysis = patient; denominators 56 and 50), magnifying endoscopy had significantly higher accuracy in predicting the nature of peritoneal lesions during surgery than white-light endoscopy (47/56, 83.93% vs. 23/50, 46.00%; χ^2^ = 16.943, *p* < 0.001), required fewer biopsies (4.0 ± 1.4 vs. 5.0 ± 1.7, *t* = −3.215, *p* = 0.002), and achieved a higher histological diagnosis rate (88.07% ± 13.84% vs. 60.06% ± 17.33%, *t* = 9.239, *p* < 0.001), with no significant difference in the number of biopsies with positive pathology (3.5 ± 1.3 vs. 3.0 ± 1.2, *p* = 0.050) (Table 2). At the lesion level (unit of analysis = lesion; denominators 77 and 68), the crude accuracies were 66/77 (85.71%) for magnifying endoscopy and 40/68 (58.82%) for white-light endoscopy; the between-group difference was significant (χ^2^ = 13.280, *p* < 0.001) and remained significant after GEE correction for within-patient clustering (Section 3.3.1).

#### 3.3.1. GEE Corrected Results—Retrospective Cohort: Magnifying Endoscopy vs. White-Light Endoscopy

Using patient ID as the subject variable and an exchangeable correlation matrix, GEE binary logistic regression was performed on 145 lesions in the retrospective cohort (77 in the magnifying endoscopy group and 68 in the white-light endoscopy group). With correct prediction (1)/incorrect prediction (0) as the dependent variable and endoscopic mode (magnifying endoscopy = 1, white-light endoscopy = 0) as the independent variable, the corrected OR was 5.646 (95% CI: 3.152–10.111), *p* < 0.001, indicating that even after fully accounting for the correlation of multiple lesions within the same patient, the intraoperative diagnostic accuracy of magnifying endoscopy remained significantly higher than that of white-light endoscopy.

#### 3.3.2. Patient-Level Sensitivity Analysis

This analysis is a patient-level sensitivity analysis with a strict aggregation criterion and should not be interpreted as confirmatory evidence of superiority. To further verify the robustness of the conclusions, a patient-level aggregation was performed using the strictest criterion: a patient was counted as “correct” only if all lesions under that patient were judged correctly; if any single lesion was judged incorrectly, the patient was counted as “incorrect.” Only 17 patients who simultaneously completed paired examinations with both magnifying endoscopy and white-light endoscopy during the same period were included. The aggregated results were as follows (Table 3):

The median number of biopsies and histological diagnosis rate at the patient level were as follows (Table 4).

At the patient level, the accuracy was 83.93% for the magnifying endoscopy group (56 patients) and 46.00% for the white-light endoscopy group (50 patients). In the patient-level analysis of 17 paired patients, the accuracy was 52.94% for magnifying endoscopy and 29.41% for white-light endoscopy. The patient-level accuracy was lower than the lesion-level accuracy, which was expected because the patient-level analysis adopted a stricter criterion. This paired analysis suggested a directional trend consistent with the lesion-level analysis; however, due to the limited sample size and lack of statistical significance, it cannot serve as independent evidence of a significant difference between the two modes. The main conclusions should still be based on the lesion-level analysis.

### 3.4. “Microvascular Classification Criteria” for Predicting the Nature of Peritoneal Lesions

**Figure 3:**
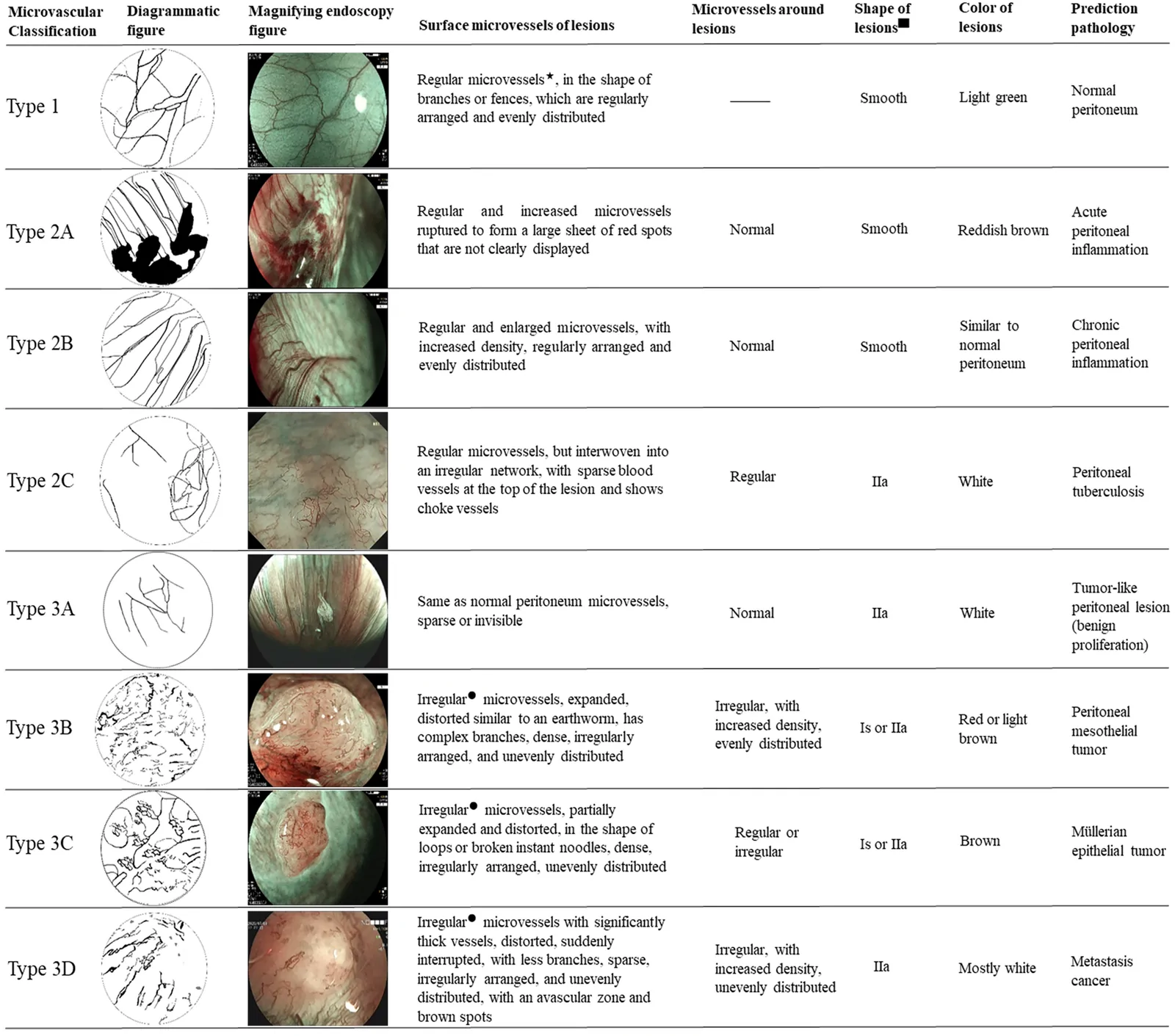
The microvascular classification criteria for predicting the nature of peritoneal lesions under the magnifying endoscopy. ^★^ Regular: Microvessels have a similar shape with uniform diameter; ^●^ Irregular: Microvessels have various shapes with diameter inconsistency, abnormal expansion (>1.5× normal); ^▀^: Shape of lesions according to Paris classification [12].

Type 1 microvessels are normal peritoneal capillaries, representing the normal peritoneum. Type 2A microvessels have smooth lesions that resemble the surrounding peritoneum with a reddish-brown color, representing acute peritoneal inflammation. Type 2B microvessels have smooth lesions that resemble the normal peritoneum, representing chronic peritoneal inflammation. Type 2C microvessels have a lesion shape of IIa(12) with a white color, representing peritoneal tuberculosis. Type 3A microvessels have a lesion shape of IIa with a white color, representing peritoneal tumor-like lesions. Type 3B microvessels have a lesion shape of Is(12) or IIa, with a white or light brown color, representing a peritoneal mesothelial tumor. Type 3C microvessels have a lesion shape of Is or IIa with a brown color, representing peritoneal Müllerian epithelial tumor. Type 3D microvessels have a lesion shape of IIa with a white color, representing peritoneal metastatic carcinoma (Figure 3).

### 3.5. Prospective Validation Results

The prospective validation cohort consecutively enrolled a total of 29 patients. After exclusion of one patient who lacked pathological confirmation, 28 patients completed the examination and were included in the study. After screening according to the exclusion criteria, 25 patients with 76 peritoneal lesions were finally included in the analysis (3 cases were reported separately as out-of-scope cases because their pathological types did not fall within the scope covered by this classification). The relationship between microvascular classification and pathological results is shown in Table 5. Among the 18 lesions classified as Type 2C, 17 were tuberculosis (94.44%) and one was metastatic carcinoma. Among the 14 lesions classified as Type 3A, 12 were tumor-like lesions (85.71%), 1 was Müllerian epithelial tumor, and one was metastatic carcinoma. Among the 13 lesions classified as Type 3B, 11 were mesothelial tumors (84.62%), 1 was tuberculosis, and one was metastatic carcinoma. Among the six lesions classified as Type 3C, 5 were Müllerian epithelial tumors (83.33%) and one was metastatic carcinoma. Among the 25 lesions classified as Type 3D, 22 were metastatic carcinoma (88.00%), 1 was tuberculosis, 1 was tumor-like lesion, and one was mesothelial tumor.

The sensitivity, specificity, PPV, NPV, and accuracy of Type 2C microvessels in predicting peritoneal tuberculosis were 89.47% (95% CI: 66.9–98.7%), 98.25% (95% CI: 90.6–100.0%), 94.44% (95% CI: 72.7–99.9%), 96.55% (95% CI: 88.1–99.6%), and 96.05% (95% CI: 88.9–99.2%), respectively. For Type 3A microvessels predicting peritoneal tumor-like lesions, these metrics were 92.31% (95% CI: 64.0–99.8%), 96.83% (95% CI: 89.0–99.6%), 85.71% (95% CI: 57.2–98.2%), 98.39% (95% CI: 91.3–100.0%), and 96.05% (95% CI: 88.9–99.2%). Type 3B microvessels in predicting peritoneal mesothelial tumors showed sensitivity, specificity, PPV, NPV, and accuracy of 91.67% (95% CI: 61.5–99.8%), 96.88% (95% CI: 89.2–99.6%), 84.62% (95% CI: 54.6–98.1%), 98.41% (95% CI: 91.5–100.0%), and 96.05% (95% CI: 88.9–99.2%), respectively. The values for Type 3C microvessels predicting peritoneal Müllerian epithelial tumors were 83.33% (95% CI: 35.9–99.6%), 98.57% (95% CI: 92.3–100.0%), 83.33% (95% CI: 35.9–99.6%), 98.57% (95% CI: 92.3–100.0%), and 97.37% (95% CI: 90.8–99.7%). Finally, Type 3D microvessels predicting peritoneal metastatic cancer exhibited sensitivity, specificity, PPV, NPV, and accuracy of 84.62% (95% CI: 65.1–95.6%), 94.00% (95% CI: 83.5–98.7%), 88.00% (95% CI: 68.8–97.5%), 92.16% (95% CI: 81.1–97.8%), and 90.79% (95% CI: 81.9–96.2%), respectively (Table 6).

#### GEE Results for Each MVP Type in the Prospective Validation Cohort

For the five MVP types (Type 2C, 3A, 3B, 3C and 3D) in the prospective validation cohort, GEE correction was also attempted. The results were as follows (Table 7):

Type 2C, 3B, 3C, and 3D presented complete or quasi-complete separation in the validation cohort, meaning that specific MVP types corresponded closely to their pathological diagnoses. Under such circumstances, the maximum likelihood estimation of GEE could not converge (singular Hessian matrix), and forcibly reporting parameter estimates based on Wald tests would produce severe bias. Therefore, for these four types, this study used the descriptive diagnostic performance indicators in Table 6 (sensitivity, specificity, accuracy, and their Clopper–Pearson exact 95% confidence intervals) as the primary validation basis.

For Type 3A predicting tumor-like lesions, because there was a small number of cross-misclassifications (1 tumor-like lesion misclassified as Type 3D, and one metastatic carcinoma misclassified as Type 3A), the GEE model successfully converged, with a corrected OR = 2.014 (95% CI: 0.421–9.647, *p* = 0.381). This OR value did not reach statistical significance, mainly due to the limited sample size of tumor-like lesions (13 lesions) and the influence of partial cross-misclassifications. However, its descriptive accuracy remained as high as 96.05%, suggesting that this type has potential clinical discriminative value but still requires validation in larger samples.

### 3.6. Strategy for Guiding the Accurate Biopsy Based on the Microvascular Classification Criteria

**Figure 4:**
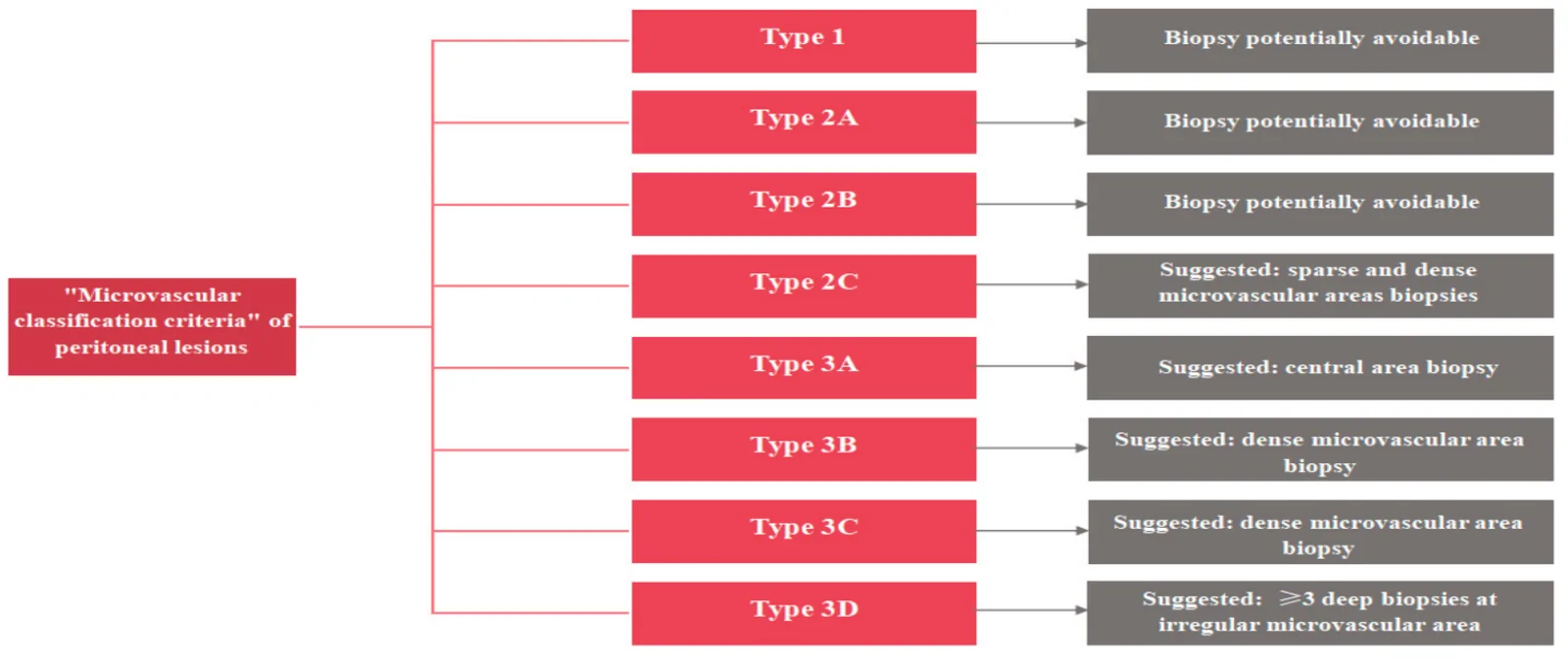
The strategy for guiding the accurate biopsy of peritoneal lesions based on the microvascular classification criteria.

According to the microvascular classification standard, corresponding exploratory precision biopsy strategies were proposed for investigational purposes (Figure 4): (1) Type 1, 2A, and 2B represent normal peritoneum, acute peritonitis, and chronic peritonitis, respectively; current evidence is insufficient to support an absolute clinical decision of “no biopsy required”; until external validation and inter-observer reproducibility have been confirmed, it is recommended that biopsy necessity be carefully evaluated in strictly selected cases. (2) Peritoneal tuberculosis is mainly classified as Type 2C according to microvascular classification. Caseous necrosis, granulomatous inflammation, and fibrous tissue areas were observed from the center to the periphery of the tuberculous nodule. No blood vessels were present in the necrotic area, resulting in the absence of microvessels at the center of the lesion under magnifying endoscopy. In the area of granulomatous inflammation, numerous inflammatory cells and newly formed small blood vessels were observed. Thus, regular microvessels similar to those in the surrounding peritoneum can be identified under magnifying endoscopy, interwoven into an irregular network. Consequently, a biopsy should be performed in both the central avascular region and marginal vascular region. (3) For tumor-like peritoneal lesions, this condition is mainly classified as Type 3A according to microvascular classification, caused by mesothelial hyperplasia, characterizing it as a non-neoplastic lesion. Microvessels observed under magnifying endoscopy resemble those of normal peritoneum, or the accumulation of proliferative mesothelial cells obscures visibility. Therefore, a biopsy should be performed in the central region. (4) Peritoneal mesothelial tumors are primarily classified as Type 3B, representing a primary peritoneal tumor. Due to the rapid proliferation of tumor cells, the blood supply increased significantly, leading to the formation of thickened new vessels. Under magnifying endoscopy, extremely dense and irregular microvessels can be identified. A biopsy should be performed in areas exhibiting dense microvessels. (5) Peritoneal Müllerian epithelial tumors are primarily classified as Type 3C. The rapid proliferation and infiltration of tumor cells necessitate increased blood supply, leading to thickened new vessels and extremely dense, irregular microvessels visible under magnifying endoscopy. A biopsy should be performed in regions with dense microvessels. (6) Peritoneal metastatic carcinoma is mainly classified as Type 3D, indicating it is a secondary tumor. The tumor tissue proliferated locally instead of diffusely infiltrating the entire lesion. Under magnifying endoscopy, thick, irregular microvessels and brown spots were observed locally within the lesion, with the remaining areas showing an avascular zone. Multiple deep biopsies should be performed in areas with irregular microvessels (≥3 times).

## 4. Discussion

### 4.1. Main Findings

This study established, for the first time, a microvascular classification standard for peritoneal lesions based on magnifying endoscopy and prospectively validated its diagnostic accuracy in a prospective cohort. The classification standard categorizes surface microvessels of peritoneal lesions into eight types, corresponding to different pathological natures, with an overall accuracy exceeding 90% in this study’s prospective cohort, which requires external validation to assess reproducibility. Meanwhile, precise biopsy strategies formulated based on this classification standard are expected to reduce false negatives caused by blind biopsy and improve the histological diagnosis rate. However, this classification standard is still in a preliminary stage, and its “good diagnostic accuracy” reflects the discriminative efficacy of the standard developers rather than universal reproducibility among physicians with different experience levels.

### 4.2. Comparison with Existing Techniques

Traditional imaging modalities (CT, MRI) struggle to identify peritoneal lesions with a diameter <1 cm or flat lesions and cannot provide a pathological diagnosis [13]. Although laparoscopic exploration allows direct visualization, the accuracy of blind biopsy based on operator experience is limited, with reported sensitivity of only 47.8% [1,2,12]. Aloisi et al. [14] proposed that NBI laparoscopy could improve the pathological positive rate of peritoneal metastasis in gynecologic cancer by assessing peritoneal lesions prior to deciding on biopsy. However, results showed that NBI laparoscopy did not provide superior detection of peritoneal surface malignancy compared to standard white-light laparoscopy, because the accuracy of predicting lesion nature based solely on large vessel morphology was limited, and NBI laparoscopy cannot clearly evaluate the microvasculature of lesions [14]. Microvessels are crucial for supplying blood and oxygen to lesions; thus, their evaluation is key to accurately determining lesion nature.

Magnifying endoscopy can display the morphology of superficial microvasculature and microstructures of lesions. Recent studies have confirmed that magnifying endoscopy can rapidly identify and assess early esophageal [15,16], gastric [8,9,17], and colorectal [10,11,18,19] cancers. This study employed magnifying endoscopy for the identification and assessment of peritoneal lesions for the first time. Results showed that magnifying endoscopy had higher crude accuracy in predicting the nature of peritoneal lesions than white-light endoscopy, required fewer biopsies, and achieved a higher histological diagnosis rate. However, this comparison has important methodological limitations: the two groups were not assigned through randomization or matching but were based on concurrent clinical pathway selection (equipment availability, operator experience, and lesion characteristics). Although available baseline data showed no significant differences between the two groups in age, sex, BMI, and major pathological type distribution, selection bias cannot be completely excluded. In addition, the total number of lesions differed between the two groups (77 vs. 68), although the mean number of lesions per patient was comparable (approximately 1.4 in both groups; *p* = 0.89); this difference in total lesion count primarily reflects the different sample sizes (56 vs. 50 patients) rather than a systematic difference in lesion burden. Therefore, this comparative result should only be regarded as an exploratory finding suggesting that magnifying endoscopy may have advantages but cannot serve as confirmatory evidence of the superiority of either mode.

Although the peritoneum and digestive tract wall have different histological features, this study demonstrates the feasibility of magnifying endoscopy in displaying peritoneal surface microvasculature, and its value in evaluating peritoneal lesions is similar to that in gastrointestinal tumors.

### 4.3. Biological Basis of Microvascular Classification

Microvascular morphology reflects the biological behavior of lesions. In non-neoplastic lesions (inflammation, tuberculosis), microvessels mostly maintain regular morphology, with uniform diameter and orderly arrangement, because although inflammatory reactions can lead to vascular proliferation, the newly formed vessels are relatively regular in morphology. In contrast, neoplastic lesions exhibit irregular microvessels with inconsistent diameter, disordered branching, and abnormally increased density due to overexpression of angiogenic factors. The local proliferation characteristics of metastatic carcinoma manifest as extremely uneven distribution of microvessels within the lesion, with avascular zones and brown spots (possibly hemorrhage or hemosiderin deposition). These differences constitute the pathophysiological basis of microvascular classification and are consistent with the microvascular patterns observed by Sano et al. [20] and Yao et al. [21] in gastrointestinal tumors.

### 4.4. Clinical Significance

The clinical significance of this study lies in: (1) providing, for the first time, a microvascular morphology-based classification standard for peritoneal lesions, filling a gap in this field; (2) focusing on small peritoneal lesions, which are the most common and most difficult to diagnose accurately in clinical practice; and (3) proposing an exploratory precise biopsy strategy requiring further validation that is expected to reduce the number of biopsies, improve the histological diagnosis rate, and avoid missed diagnoses. However, this technique requires magnifying endoscopy and NOTES equipment, places high demands on operator experience, and the learning curve remains unclear. Before wider validation and standardized training, this technique should be regarded as an investigational method evaluated only within research protocols; no recommendation for routine clinical implementation can be made at this stage. For Type 1, 2A, and 2B lesions, current evidence is insufficient to support a clinical decision of “no biopsy”; before further external validation and reproducibility confirmation, it is recommended to state that “biopsy may be avoided in strictly selected cases” rather than as an explicit clinical recommendation. In the future, artificial intelligence-assisted interpretation or simplified standards may be explored to expand applicable scenarios.

### 4.5. Limitations of the Study

This study has the following limitations:

First, as a preliminary exploratory study in this field, some subtypes (such as Type 3C) had a small number of lesions, resulting in wider 95% confidence intervals. However, the point estimate accuracy of each subtype exceeded 83%, suggesting that even with the current sample size, this classification standard demonstrates certain discriminative efficacy. Future studies should further verify its stability in larger independent cohorts.

Second, the retrospective establishment of the classification standard relied on cases with confirmed pathological diagnoses, which is a necessary prerequisite for standard development. Patients with negative biopsies were not included in this phase, primarily because they lacked a final diagnostic basis and could not be used for constructing the typing standard. The prospective validation cohort adopted a consecutive enrollment design, and the operators did not participate in the prior standard development, which largely mitigated the impact of selection bias on validation results.

Third, since some patients had multiple peritoneal lesions, lesion-level data exhibited within-group correlation. This study employed GEE correction and patient-level sensitivity analysis (re-aggregated using the strict criterion of “one error counts as all wrong”), and the results of both analytical methods were consistent in direction, suggesting that non-independence did not substantially affect the main conclusions.

Fourth, this classification standard is based on visual interpretation. During its development phase, two experienced endoscopists reached consensus through synchronous image review. This study did not conduct formal inter-observer agreement or intra-observer reproducibility assessments, nor did it invite independent physicians who did not participate in standard development to perform blinded interpretation. Therefore, it cannot be concluded that this standard has good reproducibility or clinical operability. The high accuracy observed in prospective validation reflects the discriminative efficacy of the standard developers, rather than universal reproducibility among physicians with different experience levels. In the future, stored images must be used for blinded reproducibility exercises by independent physicians with different seniority levels, and κ statistics and consistency levels must be reported before its clinical promotion value can be assessed.

Fifth, in the prospective validation phase, 3 of 29 consecutively enrolled patients (10.3%) were out-of-scope cases, suggesting that this classification standard currently covers only part of the peritoneal lesion spectrum, and its applicable boundaries need to be clarified in larger samples. In addition, although the prospective validation cohort partially overlapped with the retrospective development cohort in terms of institutions, completely independent patient populations were enrolled, and the operators in the validation phase did not participate in the prior standard development. The differences in discriminative efficacy of this standard among different lesion types suggest that its applicability in broader populations still requires further evaluation in independent multicenter cohorts.

Sixth, the diagnostic performance confidence intervals of the prospective validation cohort were calculated at the lesion level using the Clopper–Pearson method, which assumes that each lesion is an independent observation. Because the same patient might carry multiple lesions, the data exhibit within-group correlation; theoretically, the confidence intervals may be slightly narrower than those adjusted for clustering effects. Therefore, these intervals should be interpreted as descriptive references rather than strict statistical inference boundaries. If conditions permit, it is recommended to re-estimate using patient-cluster bootstrap or other cluster-correction methods.

### 4.6. Future Directions

Future research should focus on: (1) external validation in larger samples and independent centers to confirm the generalizability of the classification standard; (2) assessing inter-observer consistency among endoscopists with different experience levels, and introducing artificial intelligence-assisted image analysis if necessary to reduce operator dependence; (3) conducting cost-effectiveness analyses comparing this technology with traditional laparoscopic biopsy; (4) exploring the potential application of simplified classification standards in conventional high-definition white-light endoscopy or NBI laparoscopy to expand its clinical applicability.

## 5. Conclusions

This study integrated magnifying endoscopy with NOTES technology and established a microvascular classification standard for peritoneal lesions. This standard demonstrated good diagnostic accuracy in prospective validation and showed potential for guiding precise biopsy of peritoneal lesions, but it cannot yet serve as an independent basis for clinical decision-making. Currently, this classification standard is still in a preliminary stage, limited by the small sample size, lack of formal inter-observer consistency assessment, institutional overlap between the prospective validation cohort and the development cohort, non-random selection bias in the white light versus magnifying endoscopy comparison, and insufficient correction for lesion clustering effects in the confidence intervals. Its generalizability and reproducibility remain to be verified. Future external validation in larger independent multicenter cohorts, assessment of inter-observer consistency among physicians with different levels of experience, and clarification of the applicable boundaries of the classification standard for out-of-scope lesions should be completed before clinical application can be considered.

## Figures and Tables

**Table 1:** Characteristics of different peritoneal lesions under magnifying endoscopy.

		Pathology of Peritoneal Lesions						*p* Value ^▲^
Content		Chronic Inflammation	Tuberculosis	Tumor-like Lesion	Müllerianepithelial Tumor	Mesothelial Tumor	Metastasis Cancer	
Number of patients		7	15	10	5	6	13	
Number of lesions		9	20	12	7	7	22	
Shape of lesions	Isp	0	0	0	6 (85.71%)	1 (14.28%)	1 (4.55%)	0.000
	Is	0	2 (10.00%)	0	1 (14.29%)	3 (42.86%)	3 (13.64%)	
	IIa	3 (33.33%)	18 (90.00%)	12 (100.00%)	0	3 (42.86%)	18 (81.82%)	
	IIb	6 (66.67%)	0	0	0	0	0	
Color of lesions	White	4 (44.44%)	20 (100.00%)	9 (75.00%)	1 (14.29%)	2 (28.57%)	18 (81.82%)	0.000
	Brown	0	0	3 (25.00%)	5 (71.42%)	5 (71.43%)	4 (18.18%)	
	Same as peripheral peritoneum	5 (55.56%)	0	0	1 (14.29%)	0	0	
Boundary of lesions	Clear	2 (22.22%)	12 (60.00%)	12 (100.00%)	7 (100.00%)	7 (100.00%)	10 (45.45%)	0.000
	Unclear	7 (77.78%)	8 (40.00%)	0	0	0	12 (54.55%)	
Diameter (mm)		22.00 ± 11.50	3.13 ± 4.53	2.96 ± 2.12	9.86 ± 4.38	9.29 ± 2.98	11.68 ± 6.17	0.000
Surface of lesions	Smooth	9 (100.00%)	20 (100.00%)	12 (100.00%)	0	1 (14.29%)	6 (27.27%)	0.000
	Rough	0	0	0	7 (100.00%)	6 (85.71%)	16 (72.73%)	
Vascular around lesions	Regular	9 (100.00%)	18 (90.00%)	12 (100.00%)	3 (42.86%)	1 (14.29%)	0	0.000
	Irregular	0	2 (10.00%)	0	4 (57.14%)	6 (85.71%)	22 (100.00%)	
**Surface** **microvascular features of lesions:**								
	Visible	9 (100.00%)	20 (100.00%)	9 (75.00%)	7 (100.00%)	7 (100.00%)	22 (100.00%)	0.014
	Invisible	0	0	3 (25.00%)	0	0	0	
	Regular ^★^	9 (100.00%)	20 (100.00%)	9 (100.00%)	0	0	0	0.000
	Irregular ^●^	0	0	0	7 (100.00%)	7 (100.00%)	22 (100.00%)	
	Abnormal expansion	0	3 (15.00%)	0	7 (100.00%)	7 (100.00%)	22 (100.00%)	0.000
	No abnormal expansion	9 (100.00%)	17 (85.00%)	9 (100.00%)	0	0	0	
	Abnormal distortion	0	6 (30.00%)	1 (11.11%)	7 (100.00%)	7 (100.00%)	20 (90.91%)	0.000
	No abnormal distortion	9 (100.00%)	14 (70.00%)	8 (88.89%)	0	0	2 (9.09%)	
	Different shapes	0	0	0	7 (100.00%)	7 (100.00%)	22 (100.00%)	0.000
	Consistent shapes	9 (100.00%)	20 (100.00%)	9 (100.00%)	0	0	0	
	Diameter inconsistency	0	0	0	5 (71.43%)	7 (100.00%)	22 (100.00%)	0.000
	Uniform diameter	9 (100.00%)	20 (100.00%)	9 (100.00%)	2 (28.57%)	0	0	
	Complex branches	0	0	0	6 (85.71%)	7 (100.00%)	6 (27.27%)	0.000
	No complex branches	9 (100.00%)	20 (100.00%)	9 (100.00%)	1 (14.29%)	0	16 (72.73%)	
	Dense	6 (66.67%)	3 (15.00%)	2 (22.22%)	7 (100.00%)	7 (100.00%)	0	0.000
	Non-dense	3 (33.33%)	17 (85.00%)	7 (77.78%)	0	0	22 (100.00%)	
	Sparse	0	20 (100.00%)	6 (66.67%)	0	0	22 (100.00%)	0.000
	Non-sparse	9 (100.00%)	0	3 (33.33%)	7 (100.00%)	7 (100.00%)	0	
	Irregular arrangement	0	0	0	6 (85.71%)	7 (100.00%)	22 (100.00%)	0.000
	Regular arrangement	9 (100.00%)	20 (100.00%)	9 (100.00%)	1 (14.29%)	0	0	
	Uneven distribution	0	0	0	6 (85.71%)	7 (100.00%)	22 (100.00%)	0.000
	Even distribution	9 (100.00%)	20 (100.00%)	9 (100.00%)	1 (14.29%)	0	0	
	Choke vessels found	0	19 (95.00%)	0	0	0	22 (100.00%)	0.000
	Choke vessels not found	9 (100.00%)	1 (5.00%)	9 (100.00%)	7 (100.00%)	7 (100.00%)	0	
	Brown spots	0	0	0	0	0	17 (77.27%)	0.000
	No brown spots	9 (100.00%)	20 (100.00%)	9 (100.00%)	7 (100.00%)	7 (100.00%)	5 (22.73%)	
Nature of lesions predicted by the BLI magnification mode	Correct	7 (77.78%)	17 (85.00%)	10 (83.33%)	6 (85.71%)	7 (100.00%)	19 (86.36%)	0.930
	Incorrect	2 (22.22%)	3 (15.00%)	2 (16.67%)	1 (14.29%)	0	3 (13.64%)	
Nature of lesions predicted by the white-light mode	Correct	3 (33.33%)	16 (80.00%)	3 (25.00%)	4 (57.14%)	5 (71.43%)	9 (40.91%)	0.018
	Incorrect	6 (66.67%)	4 (20.00%)	9 (75.00%)	3 (42.86%)	2 (28.57%)	13 (59.09%)	

^★^ Regular: Microvessels have a similar shape with uniform diameter; ^●^ Irregular: Microvessels have various shapes with diameter inconsistency, abnormal expansion (>1.5× normal); ^▲^ Fisher’s exact test or Kruskal–Wallis Test.

**Table 2:** Patient-level comparison of the effectiveness of common white-light endoscopy and magnifying endoscopy in diagnosing the nature of peritoneal lesions.

Items		Magnifying Endoscopy (*n* = 56)	Common White-Light Endoscopy (*n* = 50)	χ^2^/*t* Value	*p* Value
Predicting the nature of lesions in surgery	Correct	47 (83.93%)	23 (46.00%)	16.943	0.000
	Incorrect	9 (16.07%)	27 (54.00%)		
Number of biopsy lesions (determined by operator)		4.0 ± 1.4	5.0 ± 1.7	−3.215	0.002
Number of biopsies with positive pathology		3.5 ± 1.3	3.0 ± 1.2	1.986	0.050
Positive rate of biopsy pathology (%)		88.07 ± 13.84	60.06 ± 17.33	9.239	0.000

**Table 3:** Patient-level sensitivity analysis of endoscopic diagnostic accuracy.

Endoscopic Mode	Total Patients	Patients with All Correct	Patient-Level Accuracy
Magnifying endoscopy group	17	9	52.94%
White-light endoscopy group	17	5	29.41%

Comparison between the two groups: McNemar’s test (paired design). (Note: This patient-level sensitivity analysis only included 17 patients who simultaneously completed paired examinations with both magnifying endoscopy and white-light endoscopy during the same period. The remaining 39 magnifying endoscopy patients and 33 white-light endoscopy patients without paired controls were not included in this stratified analysis.)

**Table 4:** Patient-level comparison of biopsy number and histological diagnosis rate.

Item	Magnifying Endoscopy Group (17 Patients)	White-Light Endoscopy Group (17 Patients)
Number of biopsies [median]	3	1
Histological diagnosis rate [median]	100%	57.14%

**Table 5:** Relationship between microvascular classification and the pathology of peritoneal lesions.

		Pathology of Peritoneal Lesions				
Microvascular Classification		Tuberculosis	Tumor-like Lesion	Mesothelial Tumor	Müllerian Epithelial Tumor	Metastasis Cancer
Type 2C	18	17	0	0	0	1
Type 3A	14	0	12	0	1	1
Type 3B	13	1	0	11	0	1
Type 3C	6	0	0	0	5	1
Type 3D	25	1	1	1	0	22
Total	76	19	13	12	6	26

**Table 6:** The performance of Microvascular Classification in predicting the nature of peritoneal lesions.

Microvascular Classification	Sensitivity, % (95% CI)	Specificity, % (95% CI)	PPV, % (95% CI)	NPV, % (95% CI)	Accuracy, % (95% CI)
Type 2C	89.47 (66.9–98.7)	98.25 (90.6–100.0)	94.44 (72.7–99.9)	96.55 (88.1–99.6)	96.05 (88.9–99.2)
Type 3A	92.31 (64.0–99.8)	96.83 (89.0–99.6)	85.71 (57.2–98.2)	98.39 (91.3–100.0)	96.05 (88.9–99.2)
Type 3B	91.67 (61.5–99.8)	96.88 (89.2–99.6)	84.62 (54.6–98.1)	98.41 (91.5–100.0)	96.05 (88.9–99.2)
Type 3C	83.33 (35.9–99.6)	98.57 (92.3–100.0)	83.33 (35.9–99.6)	98.57 (92.3–100.0)	97.37 (90.8–99.7)
Type 3D	84.62 (65.1–95.6)	94.00 (83.5–98.7)	88.00 (68.8–97.5)	92.16 (81.1–97.8)	90.79 (81.9–96.2)

PPV: Positive predictive value; NPV: Negative predictive value. All 95% confidence intervals were calculated using the Clopper–Pearson exact method at the lesion level.

**Table 7:** GEE results for each MVP type in the prospective validation cohort lesions.

MVP Type	Target Lesion	GEE Convergence	Result
Type 2C	Tuberculosis	Not converged	17/18 Type 2C lesions were tuberculosis, 1 was metastatic carcinoma, presenting quasi-complete separation
Type 3A	Tumor-like lesion	Converged	OR = 2.014 (95% CI: 0.421–9.647), *p* = 0.381
Type 3B	Mesothelial tumor	Not converged	11/13 Type 3B were mesothelial tumors, 1 was tuberculosis, 1 was metastatic carcinoma, presenting quasi-complete separation
Type 3C	Müllerian epithelial tumor	Not converged	5/6 Type 3C were Müllerian tumors, 1 was metastatic carcinoma, presenting quasi-complete separation
Type 3D	Metastatic cancer	Not converged	22/25 Type 3D were metastatic cancer, 1 was tuberculosis, 1 was tumor-like lesion, 1 was mesothelial tumor, presenting quasi-complete separation

## Data Availability

The original contributions presented in this study are included in the article/Supplementary Material. Further inquiries can be directed to the corresponding authors.

## Supplementary Materials

The following supporting information can be downloaded at: https://www.mdpi.com/article/10.3390/diagnostics16182984/s1, Table S1. Baseline characteristics of the retrospective comparison cohort; Table S2. Pathological subtype distribution (lesion level).

## Abbreviations

NOTES = natural orifice transluminal endoscopic surgery, CT = computed tomography, MRI = magnetic resonance imaging, BLI = blue laser imaging, NBI = narrow-band imaging, NPV = negative predictive value, OR = odds ratio, PPV = positive predictive value, SD = standard deviation, SPSS = Statistical Product and Service Solutions.

## References

[1] Levy M.J., Abu Dayyeh B.K., Fujii L.L., Clayton A.C., Reynolds J.P., Lopes T.L., Rao A.S., Clain J.E., Gleeson F.C., Iyer P.G. (2015). Detection of peritoneal carcinomatosis by EUS fine-needle aspiration: Impact on staging and resectability (with videos). Gastrointest. Endosc..

[2] Aloisi A., Sonoda Y., Gardner G.J., Park K.J., Elliott S.L., Zhou Q.C., Iasonos A., Abu-Rustum N.R. (2018). Prospective Comparative Study of Laparoscopic Narrow Band Imaging (NBI) Versus Standard Imaging in Gynecologic Oncology. Ann. Surg. Oncol..

[3] Yao K., Takaki Y., Matsui T., Iwashita A., Anagnostopoulos G.K., Kaye P., Ragunath K. (2008). Clinical application of magnification endoscopy and narrow-band imaging in the upper gastrointestinal tract: New imaging techniques for detecting and characterizing gastrointestinal neoplasia. Gastrointest. Endosc. Clin. N. Am..

[4] Schnelldorfer T., Jenkins R.L., Birkett D.H., Wright V.J., Price L.L., Georgakoudi I. (2016). Laparoscopic narrow band imaging for detection of occult cancer metastases: A randomized feasibility trial. Surg. Endosc..

[5] Sluiter N.R., Vlek S.L., Wijsmuller A.R., Brandsma H.T., de Vet H.C.W., van Grieken N.C.T., Kazemier G., Tuynman J.B. (2019). Narrow-Band Imaging Improves Detection of Colorectal Peritoneal Metastases: A Clinical Study Comparing Advanced Imaging Techniques. Ann. Surg. Oncol..

[6] Ueyama H., Yatagai N., Ikeda A., Akazawa Y., Komori H., Takeda T., Matsumoto K., Ueda K., Matsumoto K., Asaoka D. (2021). Dynamic diagnosis of early gastric cancer with microvascular blood flow rate using magnifying endoscopy (with video): A pilot study. J. Gastroenterol. Hepatol..

[7] Yamaguchi D., Kodashima S., Fujishiro M., Ono S., Niimi K., Mochizuki S., Tsuji Y., Asada-Hirayama I., Sakaguchi Y., Shichijo S. (2017). Evaluation of image-enhanced endoscopic technology using advanced diagnostic endoscopy for the detection of early gastric cancer: A pilot study. Endosc. Int. Open.

[8] Matsumura S., Dohi O., Yamada N., Harusato A., Yasuda T., Yoshida T., Ishida T., Azuma Y., Kitae H., Doi T. (2021). Improved Visibility of Early Gastric Cancer after Successful Helicobacter pylori Eradication with Image-Enhanced Endoscopy: A Multi-Institutional Study Using Video Clips. J. Clin. Med..

[9] Takayama S., Dohi O., Naito Y., Azuma Y., Ishida T., Kitae H., Matsumura S., Ogita K., Mizuno N., Terasaki K. (2021). Diagnostic Ability of Magnifying Blue Light Imaging with a Light Emitting Diode Light Source for Early Gastric Cancer: A Prospective Comparative Study. Digestion.

[10] Huang S.-L., Tan W.-X., Peng Q., Zhang W.-H., Qing H.-T., Zhang Q., Wu J., Lin L.-D., Lu Z.-B., Chen Y. (2021). Blue laser imaging combined with JNET (Japan NBI Expert Team) classification for pathological prediction of colorectal laterally spreading tumors. Surg. Endosc..

[11] Ito R., Ikematsu H., Murano T., Shinmura K., Kojima M., Kumahara K., Furue Y., Sunakawa H., Minamide T., Sato D. (2021). Diagnostic ability of Japan Narrow-Band Imaging Expert Team classification for colorectal lesions by magnifying endoscopy with blue laser imaging versus narrow-band imaging. Endosc. Int. Open.

[12] Endoscopic Classification Review Group (2005). Update on the paris classification of superficial neoplastic lesions in the digestive tract. Endoscopy.

[13] Diop A., Fontarensky M., Montoriol P.-F., Da Ines D. (2014). CT imaging of peritoneal carcinomatosis and its mimics. Diagn. Interv. Imaging.

[14] Kikuchi H., Kamiya K., Hiramatsu Y., Miyazaki S., Yamamoto M., Ohta M., Baba S., Konno H. (2014). Laparoscopic narrow-band imaging for the diagnosis of peritoneal metastasis in gastric cancer. Ann. Surg. Oncol..

[15] Ueda T., Dohi O., Naito Y., Yoshida T., Azuma Y., Ishida T., Matsumura S., Kitae H., Takayama S., Mizuno N. (2021). Diagnostic performance of magnifying blue laser imaging versus magnifying narrow-band imaging for identifying the depth of invasion of superficial esophageal squamous cell carcinoma. Dis. Esophagus.

[16] Diao W., Huang X., Shen L., Zeng Z. (2018). Diagnostic ability of blue laser imaging combined with magnifying endoscopy for early esophageal cancer. Dig. Liver Dis..

[17] Yao K., Anagnostopoulos G.K., Ragunath K. (2009). Magnifying endoscopy for diagnosing and delineating early gastric cancer. Endoscopy.

[18] Wada Y., Kudo S.-E., Kashida H., Ikehara N., Inoue H., Yamamura F., Ohtsuka K., Hamatani S. (2009). Diagnosis of colorectal lesions with the magnifying narrow-band imaging system. Gastrointest. Endosc..

[19] Sano Y., Tanaka S., Kudo S., Saito S., Matsuda T., Wada Y., Fujii T., Ikematsu H., Uraoka T., Kobayashi N. (2016). Narrow-band imaging (NBI) magnifying endoscopic classification of colorectal tumors proposed by the Japan NBI Expert Team. Dig. Endosc..

[20] Sano Y., Ikematsu H., Fu K.I., Emura F., Katagiri A., Horimatsu T., Kaneko K., Soetikno R., Yoshida S. (2009). Meshed capillary vessels by use of narrow-band imaging for differential diagnosis of small colorectal polyps. Gastrointest. Endosc..

[21] Yao K., Iwashita A., Kikuchi Y., Yao T., Matsui T., Tanabe H., Nagahama T., Sou S. (2005). Novel zoom endoscopy technique for visualizing the microvascular architecture in gastric mucosa. Clin. Gastroenterol. Hepatol..

