# Supplementary material for: Establishment and Prospective Validation of a Classification System for Surface Microvessels in Peritoneal Lesions Under Magnifying Endoscopy
Source: Diagnostics (Basel). 2026 Sep 15;16(18):2984. doi: 10.3390/diagnostics16182984 (PMC13605916; doi:10.3390/diagnostics16182984)
Supplement: Supplementary file 1 [file diagnostics-16-02984-s001.zip › diagnostics-4394022-supplementary.pdf]

Table S1. Baseline characteristics of the retrospective comparison cohort.

Characteristic	Magnifying endoscopy (n=56)	White-light endoscopy (n=50)	*P* value
Age, years	57.9 ± 14.7	53.7 ± 13.7	0.350
Sex, male/female	29/27	28/22	0.811
BMI, kg/m^2	21.6 ± 2.4	21.5 ± 2.5	0.936
Lesions per patient, n	1.4 ± 0.8	1.4 ± 0.7	0.89

Table S2. Pathological subtype distribution (lesion level)

Pathological subtype	Magnifying endoscopy (n=77 lesions)	White-light endoscopy (n=68 lesions)
Tuberculosis	20 (26.0%)	19 (27.9%)
Metastatic adenocarcinoma	22 (28.6%)	12 (17.6%)
Mesothelioma	7 (9.1%)	10 (14.7%)
Müllerian epithelial tumor	7 (9.1%)	10 (14.7%)
Tumor-like lesion	12 (15.6%)	10 (14.7%)
Chronic inflammation	9 (11.6%)	7 (10.3%)

Chi-square test for overall distribution: χ^2 = 3.914, df = 5, P = 0.5
